# Complete Chloroplast Genome Sequence of the Early Diverging Green Alga *Palmophyllum crassum*

**DOI:** 10.1128/genomeA.01745-16

**Published:** 2017-03-09

**Authors:** Ryo Furukawa, Motoshi Kunugi, Kunio Ihara, Atsushi Takabayashi, Ayumi Tanaka

**Affiliations:** a Institute of Low Temperature Science, Hokkaido University, Sapporo, Japan; b Center for Gene Research, Nagoya University, Furo-cho, Chikusa-ku, Nagoya, Aichi, Japan

## Abstract

*Palmophyllum crassum* is a little-known green alga, with a unique evolutionary position and distinctive photosynthetic features. Here, we present the complete chloroplast genome sequence of *Palmophyllum crassum*.

## GENOME ANNOUNCEMENT

*Palmophyllum crassum* is one of the earliest-branching green algae in the Tree of Life (1, 2). The *P. crassum* deep seawater habitat makes chlorophyll *b* superior to chlorophyll* a* for harvesting the available light energy present (3, 4). Therefore, *P. crassum* has evolved a unique light-harvesting system that contains large amounts of chlorophyll *b* (3). This large concentration of chlorophyll* b* is likely an adaptation to the deep-sea environment, although only a few reports on *P. crassum* photosynthetic machinery are available.

Here, we present the complete* P. crassum* chloroplast genome sequence. We have previously described *P. crassum* collection and total genomic DNA isolation (3). A total of 54,372,110 short-read sequences were generated using MiSeq technology (Illumina, CA, USA) and *de novo* assembled into 65,699 contigs using CLC Genomics Workbench version 7.5 (Qiagen, CA, USA). We were unable to *de novo* assemble any circular contigs. The contigs that did assemble contained not only chloroplast DNA but also mitochondria and nuclear DNA, because we isolated total DNA. Additionally, other organisms were likely present as contaminants, because the *P. crassum* samples were harvested from natural habitats. Therefore, we only selected contigs that showed significant tblastn (5) e values against chloroplast protein query sequences from *Mesostigma viride*, *Chlorokybus atmophyticus*, *Prasinoderma coloniale*, or *Pyramimonas parkeae* to filter out all contigs not containing *P. crassum* chloroplast DNA. Furthermore, we only selected contigs of high coverage (>100). Fifteen contigs, with sizes varying from 2,141 to 12,565 bp, were ultimately designated *P. crassum* chloroplast DNA fragments. Intercontig gaps were amplified using PCR and sequenced by the Sanger method to regenerate the intact circular chloroplast genome. Chloroplast gene annotation was primarily achieved with DOGMA (6). Additionally, tRNAscan-SE (7) was used for tRNA gene annotation. The chloroplast gene map was drawn using OGDRAW (8).

The *P. crassum* chloroplast genome is 79,397 bp in length. The genome adenine-thymine (AT) content (74.1%) is rather high among green algae, whereas the amount of intergenic sequence (16%) is somewhat lower than most green algae. No inverted repeat (IR) sequences are found in the chloroplast genome. A total of 115 genes are located in the chloroplast genome: 86 protein-coding genes, 26 tRNA genes, and three rRNA genes.

The sequence and structure of the *P. crassum* chloroplast genome are very similar to those of the recently reported chloroplast genome of *Verdigellas peltata*, which is a close relative of *P. crassum*.

### Accession number(s).

The complete chloroplast genome sequence of* P. crassum* is deposited in DDBJ/EMBL/GenBank under accession number AP017927.
